# m^6^A modification of lncRNA PHKA1‐AS1 enhances Actinin Alpha 4 stability and promotes non‐small cell lung cancer metastasis

**DOI:** 10.1002/mco2.547

**Published:** 2024-05-17

**Authors:** Qiao‐Ru Guo, Guo‐Bin Zhang, Wen‐Min Zhou, Yu Lu, Xin‐Zhu Chen, Zhuo‐Fen Deng, Jin‐Shuo Li, Hong Bi, Ming‐Sheng Wu, Ming‐Ran Xie, Yan‐Yan Yan, Jian‐Ye Zhang

**Affiliations:** ^1^ Guangzhou Municipal and Guangdong Provincial Key Laboratory of Molecular Target & Clinical Pharmacology, the NMPA and State Key Laboratory of Respiratory Disease, School of Pharmaceutical Sciences and The Fifth Affiliated Hospital Guangzhou Medical University Guangzhou P.R. China; ^2^ School of Medicine Shanxi Datong University Datong P.R. China; ^3^ Department of Pathology Shanxi Provincial People's Hospital Taiyuan P.R. China; ^4^ Department of Thoracic Surgery The First Affiliated Hospital of USTC Division of Life Sciences and Medicine University of Science and Technology of China Hefei P.R. China; ^5^ The Affiliated Qingyuan Hospital Guangzhou Medical University Qingyuan P.R. China

**Keywords:** actinin alpha 4 (ACTN4), long noncoding RNA (lncRNA), metastasis, N6‐methyladenosine (m^6^A) modification, non‐small cell lung cancer (NSCLC), phosphorylase kinase regulatory subunit alpha 1 antisense RNA 1 (PHKA1‐AS1), ubiquitination

## Abstract

Cancer is a disease with molecular heterogeneity that is closely related to gene mutations and epigenetic changes. The principal histological subtype of lung cancer is non‐small cell lung cancer (NSCLC). Long noncoding RNA (lncRNA) is a kind of RNA that is without protein coding function, playing a critical role in the progression of cancer. In this research, the regulatory mechanisms of lncRNA phosphorylase kinase regulatory subunit alpha 1 antisense RNA 1 (PHKA1‐AS1) in the progression of NSCLC were explored. The increased level of N6‐methyladenosine (m^6^A) modification in NSCLC caused the high expression of PHKA1‐AS1. Subsequently, high‐expressed PHKA1‐AS1 significantly facilitated the proliferation and metastasis of NSCLC cells, and these effects could be reversed upon the inhibition of PHKA1‐AS1 expression, both in vivo and in vitro. Additionally, the target protein of PHKA1‐AS1 was actinin alpha 4 (ACTN4), which is known as an oncogene. Herein, PHKA1‐AS1 could enhance the protein stability of ACTN4 by inhibiting its ubiquitination degradation process, thus exerting the function of ACTN4 in promoting the progress of NSCLC. In conclusion, this research provided a theoretical basis for further exploring the potential mechanism of NSCLC metastasis and searching novel biomarkers related to the pathogenesis and progression of NSCLC.

## Graphical abstract

m^6^A‐mediated lncRNA PHKA1‐AS1 promotes proliferation and metastasis of NSCLC via increasing ACTN4 stability. Increased m^6^A modification of lncRNA PHKA1‐AS1 enhanced its expression in NSCLC, which in turn regulated the expression of epithelial‒mesenchymal transition‐related proteins, leading to NSCLC metastasis. Additionally, high levels of PHKA1‐AS1 could bind to the oncogene ACTN4, inhibiting its ubiquitination degradation and enhancing its protein stability, thus promoting NSCLC progression and metastasis.

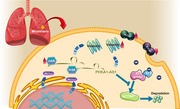

## 1 INTRODUCTION

Cancer is the leading cause of death worldwide, and it has been a topic of enduring interest and investigation in the medical field. Based on histological features, lung cancer is primarily classified as non‐small cell lung cancer (NSCLC) or small cell lung cancer, with NSCLC making up around 85% of cases. Lung adenocarcinoma (LUAD), lung squamous cell carcinoma, and large cell carcinoma are the three histological subtypes of NSCLC.^1^, ^2^ The pathogenesis of lung cancer may involve a complex combination of smoking, radon, air pollution, genetic susceptibility, radiation, diet, and other factors such as human immunodeficiency virus infection or estrogen levels.^3^ More than 70% of patients are diagnosed with advanced lung cancer, which is the main reason for the high mortality rate of lung cancer. Thus, it is imperative to further explore the etiology, early diagnosis, and treatment of lung cancer.

The transcribed genome can encode about 20,000 kinds of protein. However, the genome encoding protein only accounts for about 2% of the whole genome.^4^, ^5^ Over 200 nucleotides is the length of a kind of functional noncoding RNA (ncRNA) known as long noncoding RNA (lncRNA), which is an important regulator of gene expression, assuming a vital part in regulating biological functions and the development of various diseases, including cancer.^6^ lncRNA phosphorylase kinase regulatory subunit alpha 1 antisense RNA 1 (PHKA1‐AS1) was named by the HUGO Gene Nomenclature Committee. It was a double‐stranded RNA composed of 599 base pairs without protein‐coding capabilities. Antisense lncRNA is a special kind of lncRNA, which belongs to the natural antisense transcript with the opposite transcription direction to that encoded by protein, and widly exists in enkaryotes.^7^ lncRNA mediates gene expression regulation through a variety of mechanisms, including transcriptional regulation, post‐transcriptional regulation, translation, protein modification, acting as a molecular scaffold, and so on. Different lncRNAs play different regulatory roles in tumorigenesis, including epithelial‒mesenchymal transition (EMT) and metastasis, immune regulation, and drug resistance. Several lncRNAs such as MALAT1 have been shown to regulate the occurrence, metastasis, and resistance of NSCLC, which is concerned with the metastasis specificity of NSCLC patients.^8^, ^9^ Immune escape of tumor cells is firmly connected with the infiltration of regulatory T cells.^10^ lncRNA epidermal growth factor receptor (lnc‐EGFR) could specifically bind to tyrosine kinase receptor EGFR, thus promoting the differentiation of regulatory T cells and enhancing the immune escape function of hepatocellular carcinoma cells.^11^ Drug resistance seriously affects the efficacy of chemotherapy, molecular‐targeted therapy, and immunotherapy, ultimately leading to poor prognosis and cancer recurrence. lncRNA CASC9 was found to promote the progression of NSCLC by recruiting histone methyltransferase EZH2, inhibiting tumor suppressor DUSP1, and increasing gefitinib resistance.^12^ In addition, some lncRNAs could act as cancer suppressors, which inhibited the pathogenesis and progression of NSCLC. For example, the lncRNA FENDRR was known as a competitive endogenous RNA (ceRNA) to regulate miR‐761/TIMP2 axis, thus inhibiting the progression of NSCLC.^13^ These findings indicated that lncRNA assumed a diverse regulatory part in NSCLC, leading to an urgent demand for further research.

N6‐methyladenosine (m^6^A) modification, a dynamically reversible RNA chemical modification, is regulated by methyltransferases and demethylases. The fate of m^6^A‐modified RNAs depends on the ability to recognize and bind their different “readers,” which further affects RNA splicing, translation, stability, and interaction with protein.^14^, ^15^ The increased m^6^A modification of lncRNA can influence the expression level of lncRNA in cells, thus activating the downstream pathway and affecting the development of cancer. For instance, the m^6^A modification of lncRNA ABHD11‐AS1 induced by m^6^A methyltransferase METTL3 was increased, which could enhance the stability of the transcript, thereby up‐regulating the expression of ABHD11‐AS1 and facilitating the proliferation and Warburg effect of NSCLC.^16^ lncRNA DGUOK‐AS1 increased the stability of TRPM7 by regulating m^6^A modification mediated by METTL3/IGF2BP2, thus facilitating the growth and metastasis of NSCLC cells.^17^ m^6^A modification improved the stability of SNHG1 methylated transcript by reducing RNA degradation rate, which led to the up‐regulation of SNHG1 in NSCLC.^18^ Therefore, exploring the m^6^A modification is of great significance for studying the roles of lncRNA and the development of NSCLC.

At present, no explanation has been given about the function and mechanism of this PHKA1‐AS1 in NSCLC. It is necessary to further study this RNA molecule to reveal its function, mechanism, and potential clinical application. This research was aimed to explore the effect of PHKA1‐AS1 in the progression of NSCLC and its possible molecular mechanism. Combined with the above, we proposed the hypothesis that m^6^A modification increased the expression of PHK1‐AS1, which led to the progression of NSCLC.

## 2 RESULTS

### 2.1 PHKA1‐AS1 was highly expressed in NSCLC cells and carcinoma tissues

**FIGURE 1:**
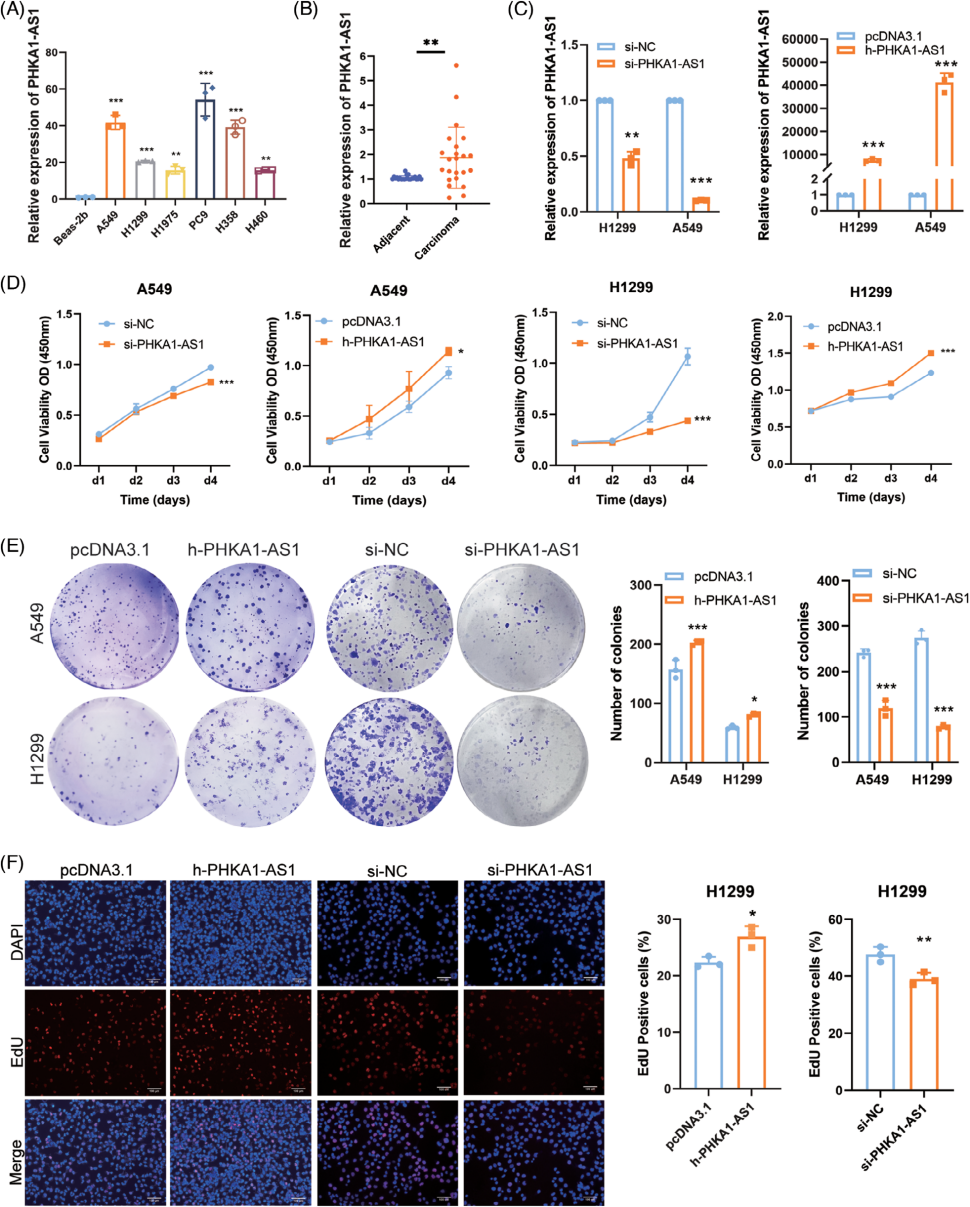
Phosphorylase kinase regulatory subunit alpha 1 antisense RNA 1 (PHKA1‐AS1) exhibited high expression levels in non‐small cell lung cancer (NSCLC) cells and influenced cell viability. (A) Comparison of expression levels of PHKA1‐AS1 among NSCLC cell lines and Beas‐2b cells. ^**^ *p* < 0.01, ^***^ *p* < 0.001, *n* = 3. (B) Expression of PHKA1‐AS1 in carcinoma tissues and adjacent tissues of patients with NSCLC. ^**^ *p* < 0.01, *n* = 23. (C) The transfection efficiency of up‐regulating and down‐regulating PHKA1‐AS1. ^**^ *p* < 0.01, ^***^ *p* < 0.001, *n* = 3. (D) Absorbance changes of cell counting kit‐8 (CCK‐8) for four consecutive days after transfection. ^*^ *p* < 0.05, ^***^ *p* < 0.001, *n* = 3. (E) Verification of colony forming ability of NSCLC cells in vitro by colony formation assay. ^*^ *p* < 0.05, ^***^ *p* < 0.001, *n* = 3. (F) Verification of proliferation ability of NSCLC in vitro by 5‐ethyny‑2′‑deoxyuridine (EdU) assay (scale bar = 100 µm). ^*^ *p* < 0.05, ^**^ *p* < 0.01, *n* = 3.

To verify the specific expression of PHAK1‐AS1 in NSCLC cells, total RNA from human normal lung epithelial cell line Beas‐2b and NSCLC cell lines, including A549, H1299, H1975, PC9, H358, and H460, was extracted. In addition, carcinoma samples and adjacent tissues from 23 NSCLC patients were included. By using qPCR to evaluate the endogenous expression level of PHKA1‐AS1, it was possible to determine that NSCLC cells expressed PHKA1‐AS1 at a higher level than Beas‐2b cells (Figure 1A). In a similar vein, PHKA1‐AS1 expression in cancer tissues was much higher than adjacent tissues (Figure 1B). The impact of PHKA1‐AS1 expression level on NSCLC cell viability was next investigated utilizing the cell counting kit‐8 (CCK‐8) assay. Four days after transfection was used to test cell viability, and the results indicated that up‐regulating PHKA1‐AS1 improved NSCLC cell viability, whereas the opposite result was obtained after down‐regulating PHKA1‐AS1 (Figure 1C, D). Then, the impact of PHKA1‐AS1 on the in vitro proliferation of NSCLC cells was detected by colony formation assay and 5‑ethyny‑2′‑deoxyuridine (EdU) assay. After up‐regulating PHKA1‐AS1, the number and size of A549 and H1299 cell colonies were larger than those in negative control (NC) group, while smaller after down‐regulating PHKA1‐AS1 (Figure 1E). Similarly, the EdU‐positive cell rate was higher than that in the NC group after up‐regulating PHKA1‐AS1 and lower than NC group after down‐regulating PHKA1‐AS1 (Figure 1F). The results indicated that high‐expressed PHKA1‐AS1 could promote the proliferation of NSCLC cells in vitro.

### 2.2 PHKA1‐AS1 promoted the migration and invasion of NSCLC cells in vitro

**FIGURE 2:**
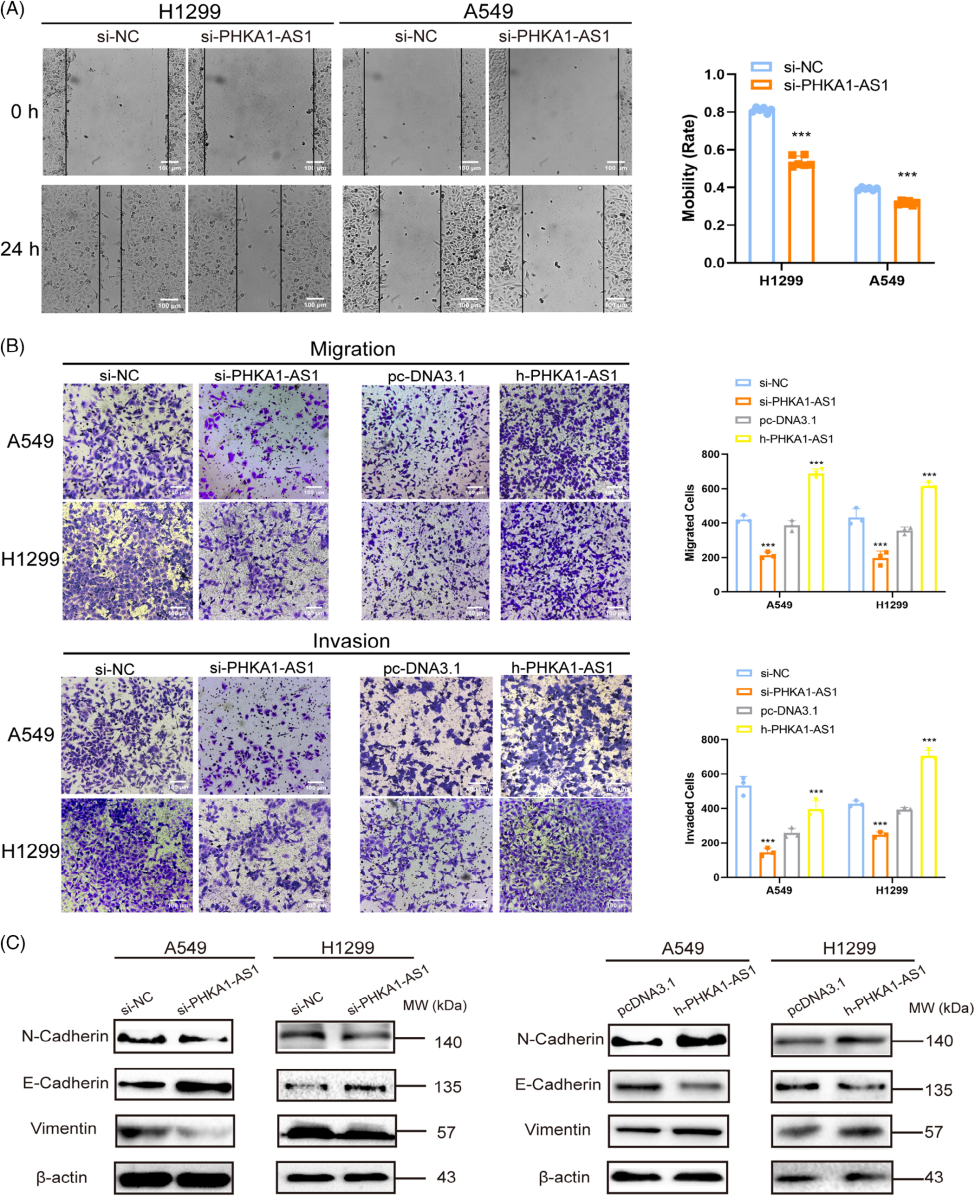
Effects of phosphorylase kinase regulatory subunit alpha 1 antisense RNA 1 (PHKA1‐AS1) on the migration and invasion of non‐small cell lung cancer (NSCLC) cells. (A) The effect of PHKA1‐AS1 on plane migration of A549 and H1299 cells (scale bar = 100 µm). ^***^ *p* < 0.001, *n* = 6. (B) The effect of PHKA1‐AS1 on the space migration and invasion of A549 and H1299 cells (scale bar = 100 µm). ^***^ *p* < 0.001, *n* = 3. (C) The regulatory effect of PHKA1‐AS1 on epithelial‒mesenchymal transition (EMT) markers by Western blotting assay.

The impact of PHKA1‐AS1 on the migration and invasion of NSCLC cells in vitro was determined by wound healing assay and transwell assay. Transwell chambers pre‐coated with Matrigel was applied to verify the invasion ability of NSCLC cells, while uncoated chambers were used to verify the migration ability. The results of wound healing assay indicated that the migration capacity of H1299 and A549 cell lines was inhibited after down‐regulating PHKA1‐AS1 (Figure 2A). The results of transwell assay demonstrated that high‐expressed PHKA1‐AS1 facilitated, while low‐expressed PHKA1‐AS1 inhibited the A549 and H1299 cells from migrating and invading (Figure 2B). Then, Western blotting assay displayed that low‐expressed PHKA1‐AS1 could up‐regulate E‐cadherin but down‐regulate N‐cadherin and Vimentin expression in A549 and H1299 cells, while high‐expressed PHKA1‐AS1 could down‐regulate E‐cadherin but promote N‐cadherin and Vimentin expression (Figure 2C). Consequently, PHKA1‐AS1 possessed the ability to facilitate NSCLC cells to migrate and invade in vitro.

### 2.3 PHKA1‐AS1 was located in the cytoplasm and bound to ACTN4

**FIGURE 3:**
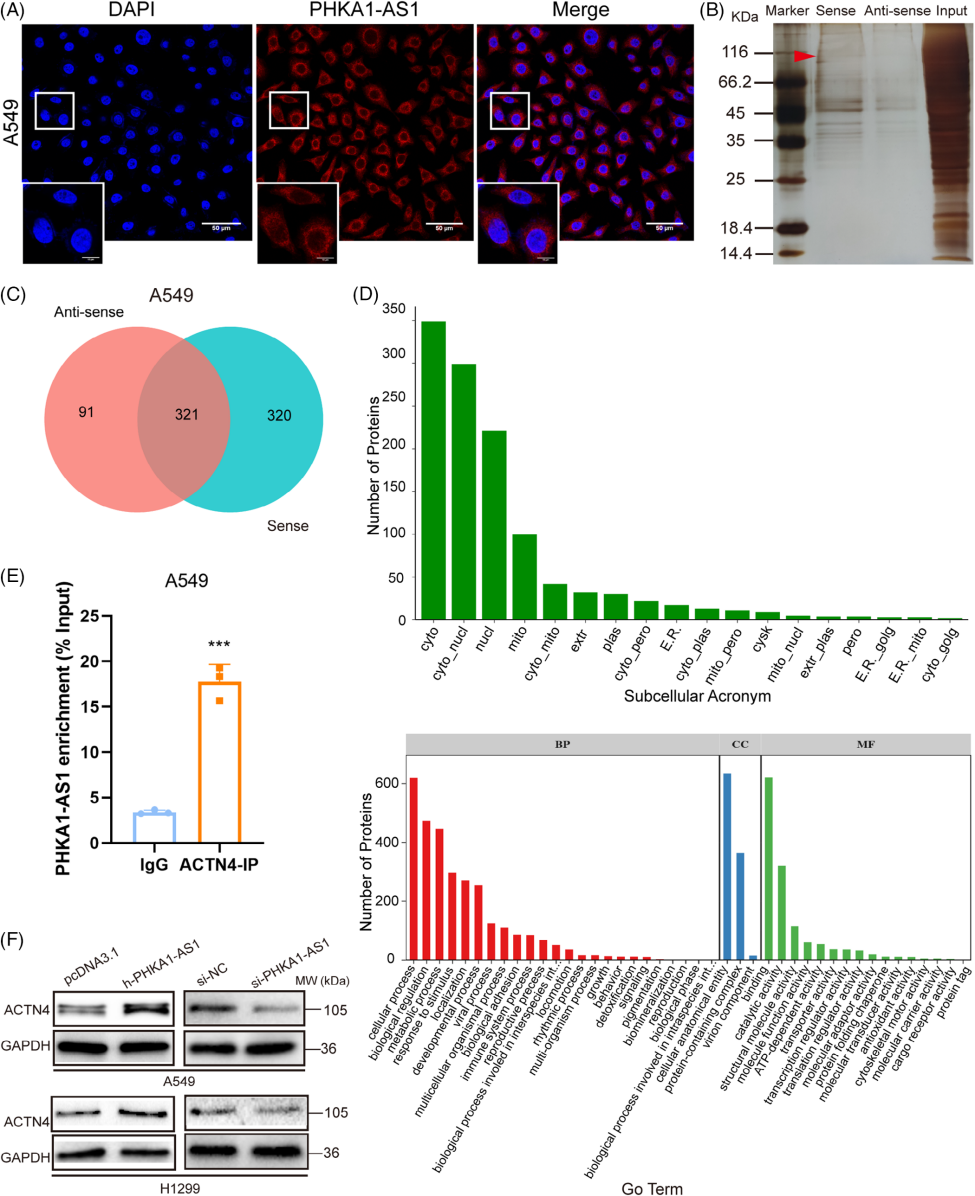
Phosphorylase kinase regulatory subunit alpha 1 antisense RNA 1 (PHKA1‐AS1) was found to localize in the cytoplasm and interact with actinin alpha 4 (ACTN4). (A) PHKA1‐AS1 and cell nucleus were labeled with PHKA1‐AS1 specific fluorescent probe (red) and Hoechst33342 dye (blue), respectively. The subcellular localization of PHKA1‐AS1 was detected by fluorescence in situ hybridization (FISH) assay (scale bar for large photos = 50 µm; scale bar for small photos = 10 µm). (B) Silver staining was performed on the RNA pull‐down protein (red arrow: position of target protein ACTN4). (C) After mass spectral analysis of the pull‐down proteins in the anti‐sense group and the sense group, the intersecting Venn diagram was taken. (D) Subcellular distribution analysis (https://wolfpsort.hgc.jp/) of RNA pull‐down proteins and functional annotation of protein Gene Ontology (GO). (E) The interaction between PHKA1‐AS1 and ACTN4 was demonstrated by RNA immunoprecipitation (RIP) assay. ^***^ *p* < 0.001, *n* = 3. (F) Western blotting assay was used to detect the expression of ACTN4 after up‐regulating or down‐regulating PHKA1‐AS1.

The function of lncRNA is closely related to its subcellular localization. Exploring the subcellular localization of lncRNA and its local interaction with molecules is the key to predict its function.^19^ Fluorescence in situ hybridization (FISH) localization of PHKA1‐AS1 in A549 cells was performed by FISH assay, and the findings showed that the location of PHKA1‐AS1 was in the cytoplasm (Figure 3A). Using RNA pull‐down assay combined with protein spectrum detection, hundreds of proteins that bound to PHKA1‐AS1 were found (Figure 3B). After searching and analyzing by software Proteinpilot, when the confidence level was more than 95% and at least contained a unique peptide, the total numbers of proteins identified by antisense and sense group were 412 and 641, respectively. A total of 321 proteins were identified in two samples, with 91 and 320 unique proteins identified in the antisense and sense groups, respectively (Figure 3C). Subsequently, WoLFPSORT (https://wolfpsort.hgc.jp/) was utilized to anticipate the subcellular localization of these proteins, and protein Gene Ontology function analysis was performed to preliminarily infer the mechanism by which PHKA1‐AS1 regulates biological functions. These findings demonstrated that the majority of PHKA1‐AS1 binding proteins were localized in the cytoplasm, and the biological processes of binding proteins was related to cellular processes, biological regulation, and metabolic processes, and the molecular function involved were related to binding, catalytic activity and structural molecular activity. (Figure 3D). According to the results of the protein spectrum, the high specificity of binding was found between ACTN4 and PHKA1‐AS1. The binding effect was verified by RNA immunoprecipitation (RIP) assay, and the result showed that ACTN4 could pull down PHKA1‐AS1 in A549 cells (Figure 3E). Later, the regulating effect of PHKA1‐AS1 and ACTN4 was verified by Western blotting assay. High expression of PHKA1‐AS1 up‐regulated the ACTN4 expression, while low‐expressed PHKA1‐AS1 down‐regulated the ACTN4 expression (Figure 3F).

### 2.4 ACTN4 promoted the proliferation, migration, and invasion of NSCLC

**FIGURE 4:**
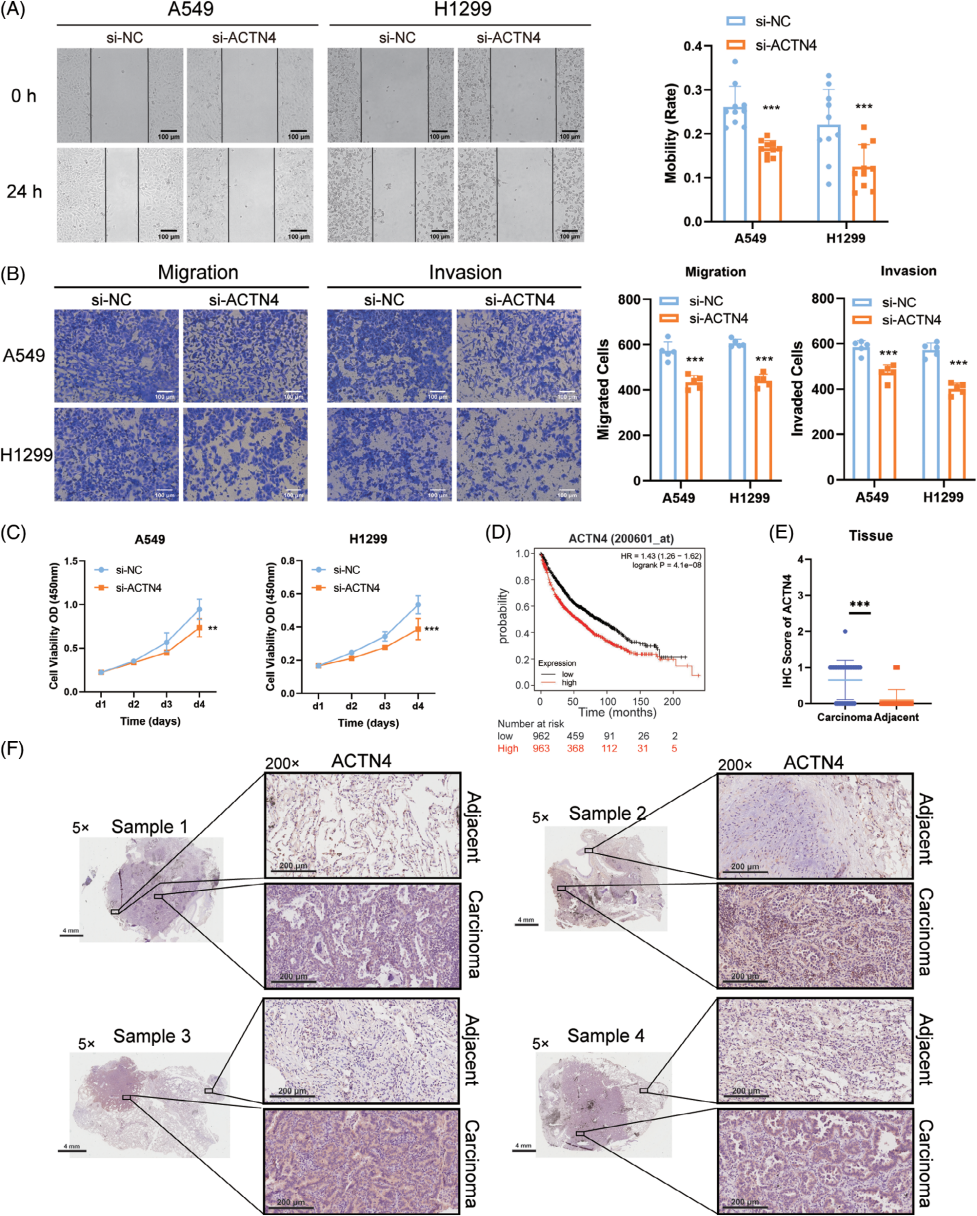
Actinin alpha 4 (ACTN4) facilitated the progression of non‐small cell lung cancer (NSCLC). (A) The effect of ACTN4 on plane migration of A549 and H1299 cells (scale bar = 100 µm). ^***^ *p* < 0.001, *n* = 10. (B) The effect of ACTN4 on space migration and invasion of A549 and H1299 cells (scale bar = 100 µm). ^***^ *p* < 0.001, *n* = 5. (C) Absorbance changes of cell counting kit‐8 (CCK‐8) for four consecutive days after down‐regulating ACTN4. ^**^ *p* < 0.01, ^***^ *p* < 0.001, *n* = 3. (D) Survival curve between the expression of ACTN4 and the survival rate of patients with NSCLC (http://kmplot.com/analysis/). (E) Statistics of the immunohistochemistry (IHC) score of ACTN4. ^***^ *p* < 0.001, *n* = 32. (F) Representative images of IHC of ACTN4 (scale bar for 5× images were 4 mm; scale bar for 200× images were 200 µm).

Studies have shown that ACTN4, one of the actin‐binding family, was a non‐actin protein associated with tumor development, invasion, and metastasis.^20^ Here, wound healing assay and transwell migration and invasion assay were utilized to detect the function of ACTN4 in NSCLC cells. As shown in the results, the NSCLC cells could be inhibited from proliferating, migrating, and invading by knocking down ACTN4 (Figure 4A‒C). When comparing lung cancer patients with low expression of ACTN4 to those with high expression, the Kaplan‒Meier Plotter database (http://kmplot.com/analysis/) revealed that the former had a better prognosis and a longer survival period (Figure 4D). Accordingly, the expression of ACTN4 in carcinoma tissues was higher than that in adjacent tissues in patients with NSCLC, which was detected by immunohistochemistry (IHC) assay (Figure 4E,F). These results suggested that ACTN4 was significantly expressed and aided in the disease's progression in NSCLC.

### 2.5 PHKA1‐AS1 inhibited the ubiquitination degradation of ACTN4

**FIGURE 5:**
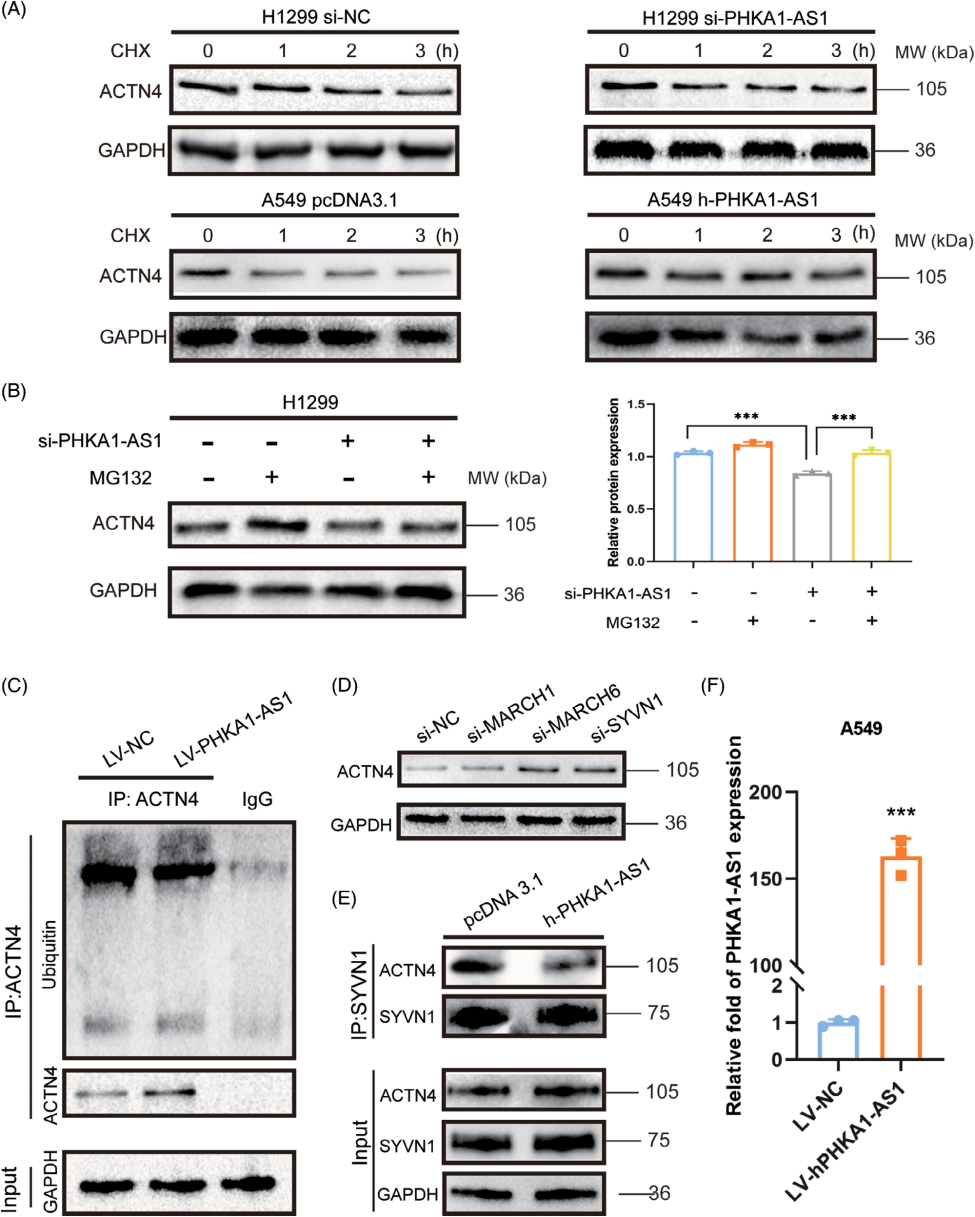
Phosphorylase kinase regulatory subunit alpha 1 antisense RNA 1 (PHKA1‐AS1) was involved in regulating the ubiquitination degradation pathway of actinin alpha 4 (ACTN4). (A) The effect of PHKA1‐AS1 on the stability of ACTN4 was detected by Western blotting assay. (B) The ability of PHKA1‐AS1 and MG132 to regulate ACTN4 degradation was demonstrated by Western blotting assay. ^***^ *p* < 0.001, *n* = 3. (C) The polyubiquitination level of ACTN4 after up‐regulating PHKA1‐AS1. (D) Detection of ACTN4 expression after knockdown of E3 ubiquitin ligase. (E) Binding ability of ACTN4 to E3 ubiquitin ligase SYVN1 after up‐regulating PHKA1‐AS1. (F) Transfection efficiency of A549‐PHKA1‐AS1 stably transfected cell line constructed by lentivirus. ^***^ *p* < 0.001, *n* = 3.

The binding of lncRNA to protein can affect the stability of the binding protein.^21^ The result of protein stability assay showed that the degradation rate of ACTN4 in high‐expressed PHKA1‐AS1 group was slower than NC group, while the degradation rate of ACTN4 in si‐PHKA1‐AS1 group was faster than NC group (Figure 5A). These findings demonstrated that PHKA1‐AS1 could enhance the stability of ACTN4. Subsequently, the result showed that MG132 could significantly reverse the down‐regulation of ACTN4 expression caused by low‐expressed PHKA1‐AS1 (Figure 5B). It suggested that PHKA1‐AS1 might affect the stability of ACTN4 by regulating its proteasomal pathway. Then, protein co‐immunoprecipitation (Co‐IP) assay displayed that high‐expressed PHKA1‐AS1 could significantly reduce the polyubiquitination level of ACTN4 (Figure 5C,F). Later, E3 ubiquitin ligases, including MARCH1, MARCH6, and SYVN1, were predicted to most likely bind to ACTN4 using UbiBrowser 2.0 database (http://ubibrowser.ncpsb.org.cn) (Figure S1). When these three genes were knocked down separately in NSCLC cells, the expression of ACTN4 in the cells was up‐regulated, including the most significant change in si‐SYVN1 group (Figure 5D). Then, Co‐IP assay showed that after up‐regulating PHKA1‐AS1, the binding ability of SYVN1 to ACTN4 was reduced (Figure 5E). It could be deduced that PHKA1‐AS1 was able to inhibit the ubiquitination degradation by inhibiting the binding between ACTN4 and E3 ubiquitin ligase SYVN1, thus enhancing the stability of ACTN4.

### 2.6 m^6^A modification promoted the expression of PHKA1‐AS1

**FIGURE 6:**
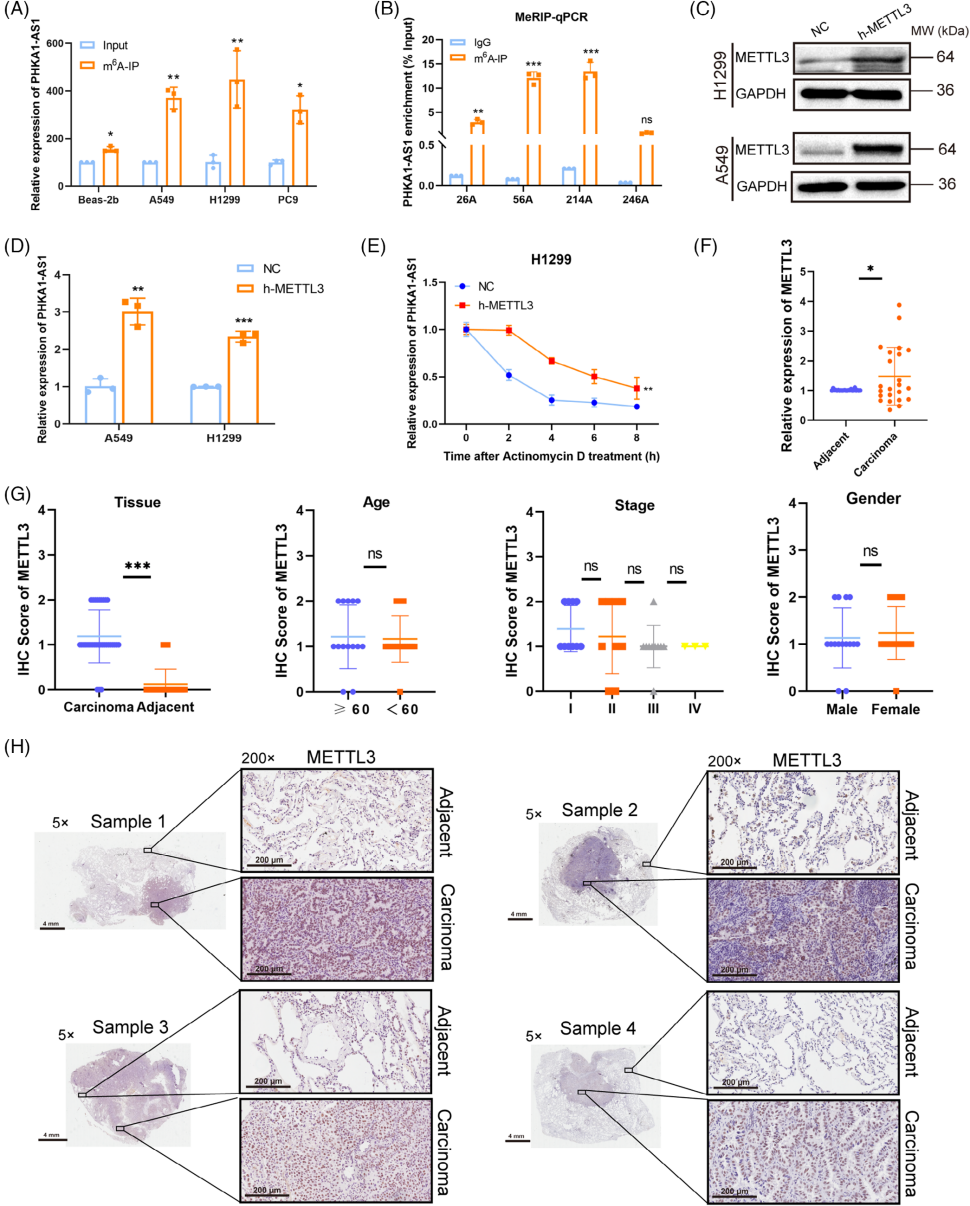
The expression of phosphorylase kinase regulatory subunit alpha 1 antisense RNA 1 (PHKA1‐AS1) was promoted by N6‐methyladenosine (m^6^A) modification. (A) The m^6^A‐RNA immunoprecipitation (RIP) assay was used to detect the m^6^A modification of PHKA1‐AS1 in Beas‐2b, A549, H1299, and PC9 cells, respectively. ^*^ *p* < 0.05, ^**^ *p* < 0.01, *n* = 3. (B) m^6^A RNA immunoprecipitation (MeRIP)‐qPCR showed that m^6^A modification of PHKA1‐AS1 occurred at 26A, 56A, and 214A sites. ns: no significance, ^**^ *p* < 0.01, ^***^ *p* < 0.001, *n* = 3. (C) The transfection efficiency of METTL3 was verified by Western blotting assay. (D) qPCR assay was used to detect the expression of PHKA1‐AS1 after up‐regulating METTL3 in A549 and H1299 cells. ^**^ *p* < 0.01, ^***^ *p* < 0.001, *n* = 3. (E) Expression of PHKA1‐AS1 in NC group and over‐expressed METTL3 group after actinomycin D treatment in H1299 cells. ^**^ *p* < 0.01, *n* = 3. (F) Expression of METTL3 in carcinoma tissues and adjacent tissues of patients with NSCLC. ^*^ *p* < 0.05, *n* = 23. (G) Statistics of the immunohistochemistry (IHC) score level of the sections. ns: no significance, ^***^ *p* < 0.001, *n* = 32. (H) Representative images of IHC of METTL3 (scale bar for 5× images was 4 mm; scale bar for 200× images was 200 µm).

The m^6^A modification regulates RNA functions by regulating all stages of the RNA lifecycle, such as RNA processing, translation, and nuclear output.^22^ Here, m^6^A‐RIP assay was utilized to determine the m^6^A modification level of PHKA1‐AS1 in NSCLC cell lines and Beas‐2b cells. As shown in the results, the m^6^A modification level of PHKA1‐AS1 in A549, H1299, and PC9 cells was increased significantly, compared with Beas‐2b cells (Figure 6A). Later, using SRAMP algorithm (http://www.cuilab.cn/sramp), seven potential sites (26A, 40A, 56A, 182A, 214A, 246A, 322A) of PHKA1‐AS1 were identified (Figure S2). Among them, 26A, 56A, 214A, and 246A were the top four sites of confidence score. Subsequently, m^6^A RNA immunoprecipitation (MeRIP) coupled qPCR assay demonstrated that m^6^A modification could occur at 26A, 56A, and 214A sites, and the modification degree of 56A and 214A was most significant (Figure 6B). Methyltransferase METTL3 was an important catalytic enzyme, which could cause m^6^A methylation modification of mRNA. When METTL3 was over‐expressed, PHKA1‐AS1 expression was likewise elevated in NSCLC cells (Figure 6C,D). In addition, actinomycin D (5 µg/mL) was applied to cells for 0, 2, 4, 6, and 8 h after METTL3 was up‐regulated, and then qPCR assay was used to detect the level of PHKA1‐AS1 in H1299 cells. The results showed that up‐regulated METTL3 significantly slowed down the degradation rate of PHKA1‐AS1 (Figure 6E). Besides, the results of qPCR and IHC showed that patients with NSCLC exhibited far greater levels of METTL3 expression in their carcinoma tissues than in adjacent tissues (Figure 6F‒H). These results suggested that PHKA1‐AS1 was regulated by m^6^A modification and thus highly expressed in NSCLC.

### 2.7 Down‐regulated PHKA1‐AS1 could inhibit the lung metastasis of NSCLC cells in vivo

**FIGURE 7:**
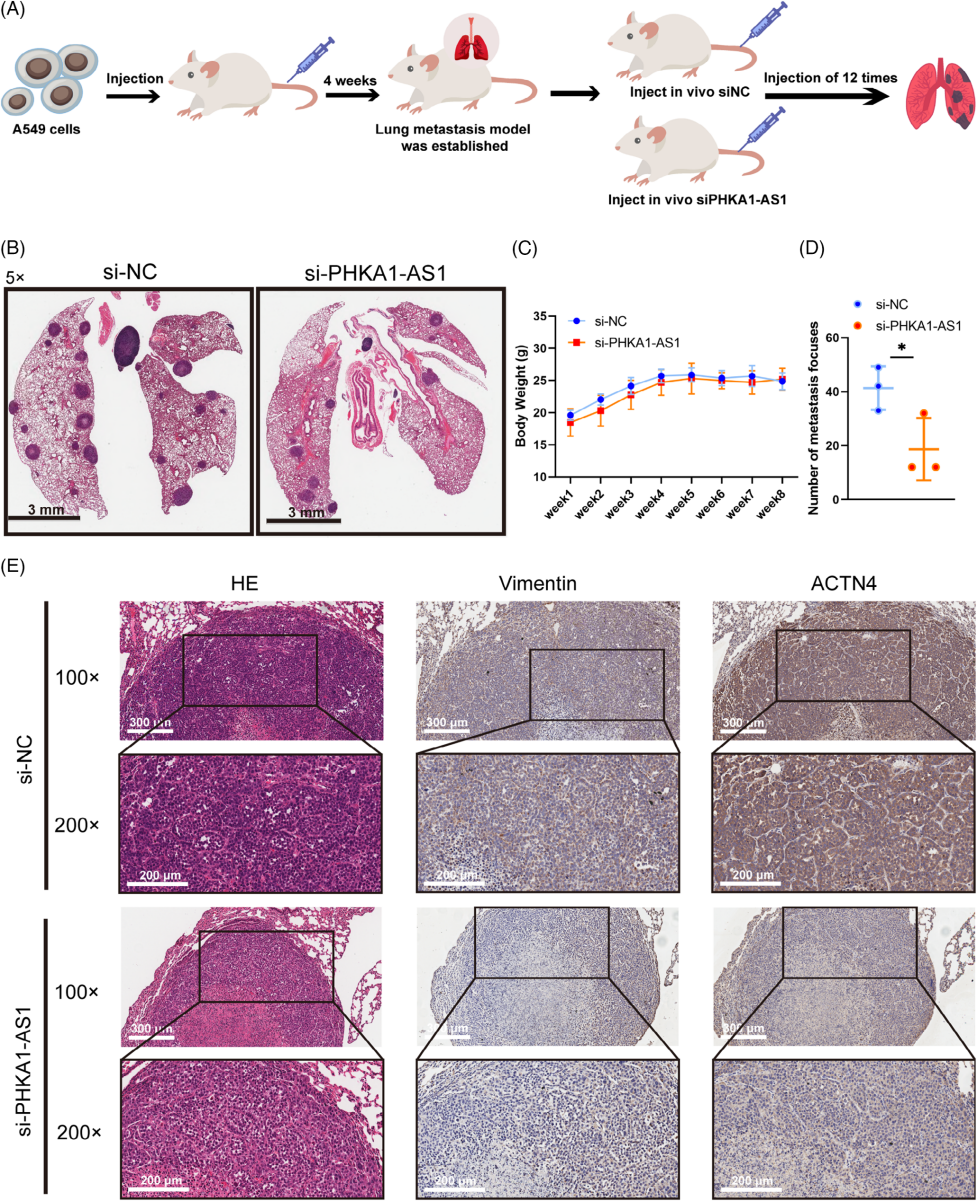
Phosphorylase kinase regulatory subunit alpha 1 antisense RNA 1 (PHKA1‐AS1) influenced lung metastasis of non‐small cell lung cancer (NSCLC) in vivo. (A) Overview of the establishment of lung metastasis model and experiment operating process in nude mice. (B) Hematoxylin/eosin (HE) staining of the whole lung of nude mice, panoramic section showed the number of lung metastases (scale bar: 3 mm). (C) Weight growth curve of nude mice. (D) Statistics of the number of lung metastases in nude mice. ^*^ *p* < 0.05, *n* = 3. (E) Representative images of HE staining and immunohistochemistry (IHC) (scale bar for 100× images was 300 µm; scale bar for 200× images was 200 µm).

To further explore the effect of PHKA1‐AS1 on lung metastasis of NSCLC, Balb/c‐nude mice model of lung metastasis by tail vein injection of A549 cells was established. Four weeks after cell injection, two groups of nude mice received an injection of 5 nmol si‐NC and 5 nmol si‐PHKA1‐AS1 into their tail veins every 2 days for a total of 12 times (Figure 7A). The body weights of the nude mice were recorded weekly for 8 weeks (Figure 7C). After the nude mice were dissected, the results displayed that lung metastasis in the si‐PHKA1‐AS1 group was fewer than those in the si‐NC group (Figure 7B,D). The expression of Vimentin and ACTN4 was found to be down‐regulated in lung metastasis tumors after down‐regulating PHKA1‐AS1 using IHC assay (Figure 7E). The results indicated that reducing the expression of PHKA1‐AS1 may prevent NSCLC cells from metastasizing in vivo.

## 3 DISCUSSION

Cancer is the main disease that threatens human health, and lung cancer is the leading cause of cancer‐related death globally. Despite significant advancements in medical technology for the diagnosis and treatment of NSCLC, the 5‐year survival rate for the condition remains low. As a result, it is still necessary to further explore the pathogenesis, early diagnosis, and treatment of NSCLC. ncRNA accounts for about 90% of RNA transcribed from the human genome. Over the past decade, people have gradually realized that ncRNA regulatory network assumed a vital part in the occurrence and development of cancer. ncRNA can affect several aspects of cancer development, including EMT, metastasis, proliferation, and immune regulation. As a reversible RNA modification, m^6^A is regulated by three groups of enzymes, including methyltransferase (Writer), such as METTL3 and METTL14; demethylases (Eraser), such as ALKBH5 and FTO; and unscrambling enzyme (Reader), such as YTHDF1 and YTHDF2.^23^ Modification of lncRNA by m^6^A can affect the splicing and maturation of lncRNA, structural changes, stability, and so on, thus affecting the development of cancer through its complex molecular regulatory. Therefore, further exploration of the effect of m^6^A‐modified lncRNA on cancer can help us deepen our understanding of the pathogenesis of cancer and offer a strong theoretical foundation for investigating possible cancer targets for diagnosis, prognosis, and treatment.

The process of tumor metastasis is a stepwise cascade event. Literature has shown that lncRNA was involved in the metastatic cascade of cancer and could induce molecular or phenotypic changes in cancer cells.^24^ Dysregulation of lncRNAs contributes to tumorigenesis and metastasis in different ways. For example, lncRNA MAFG‐AS1 was abnormally expressed in many cancers, which affects cell proliferation and metastasis by regulating various cell signaling pathways, including EMT.^25^ During the EMT process, the decrease in the epithelial marker E‐cadherin and the increase in mesenchymal marker N‐cadherin or Vimentin could gradually transform the epithelial‐like characteristics into mesenchymal‐like characteristics with greater invasiveness and motility.^26^ Herein, PHKA1‐AS1 was found highly expressed in NSCLC cells, and facilitated proliferation, migration, and invasion of NSCLC in vitro (Figures 1 and 2). Accordingly, knocking down PHKA1‐AS1 could inhibit the metastasis of NSCLC in nude mice (Figure 7). These results initially verified the ability of PHAK1‐AS1 to promote tumor metastasis in vitro and in vivo.

The function of lncRNA is closely related to its subcellular localization. The lncRNA located in the nucleus is often used as a chromatin regulator to regulate transcription program by interacting with chromatin and chromatin remodeling, thereby affecting gene expression.^27^ The cytoplasmic lncRNA generally functions as a protein scaffold to influence the expression of target genes by absorbing RNA‐binding proteins^28^; or absorbing the miRNA by ceRNA mechanism to affect the stability and activity of the mRNA^29^; or directly combining with protein to regulate post‐translational modification of the protein.^30^ This study indicated that PHKA1‐AS1 was mainly localized in the cytoplasm (Figure 3). Therefore, its mechanism might be related to the interaction between lncRNA and protein in the cytoplasm, so as to play the regulatory role in NSCLC. Herein, high specificity binding of ACTN4 to PHKA1‐AS1 based on the results of the RNA pull‐down assay and protein spectrum was found. Subsequently, Western blotting assay and RIP assay verified the binding effect of PHKA1‐AS1 and ACTN4. High‐expressed PHKA1‐AS1 showed the promotion effect on ACTN4 expression, while low‐expressed PHKA1‐AS1 displayed the opposite consequence (Figure 3). As a member of the α‐actinin family of actin crosslinking proteins, ACTN4 was up‐regulated in several types of cancer and implicated in the metastasis of cancer.^31^ For example, ACTN4 promoted the activation of Akt, which led to the up‐regulation of Snail expression and then regulated the expression of E‐cadherin.^20^ Later, Snail‐mediated matrix metalloproteinase‐9 expression promoted cell migration and invasion. Besides, ACTN4 inhibited the proteasome degradation of β‐catenin in Akt/GSK‐3β‐dependent manner, thus regulating the stability of β‐catenin.^32^ These genes regulated by ACTN4 were vital in EMT, which led to tumor progression. In this study, ACTN4 was found highly expressed in NSCLC. Down‐regulation of ACTN4 resulted in inhibition of proliferation, migration, and invasion of NSCLC cells (Figure 4).

To investigate the molecular mechanism of PHKA1‐AS1 in regulating ACTN4, further experiments were conducted. In the process of protein modification, ubiquitin‒proteasome degradation pathway is one of the most important mechanisms to control protein expression level. Ubiquitin, a protein evolving to be conserved, may post‐translationally mark proteins for destruction. Ubiquitin molecules are transported to ubiquitin‐conjugating enzyme E2 after being activated by ubiquitin‐activating enzyme E1, where they are then transferred to protein substrate by E3.^33^ It has been mentioned that lncRNA is crucial to this procedure, and thus regulates the proliferation, metastasis, and apoptosis of tumor.^33^, ^34^ Therefore, we sought to reveal the mechanism of PHKA1‐AS1 in promoting tumor progression by exploring its ability to regulate the ubiquitination and degradation process of ACTN4. As shown in the results, PHKA1‐AS1 inhibited the ubiquitination and degradation pathway of ACTN4 by inhibiting the binding of E3 ligase SYVN1 to ACTN4 (Figure 5).

The methylation modification of m^6^A can regulate the expression level of lncRNA, thus affecting the downstream pathway and regulating the development of cancer.^35^ The most significant protein in the complex that catalyzes the m^6^A alteration of RNA molecules on N6‐methyladenine is METTL3. This complex is known as the m^6^A methyltransferase complex.^36^ m^6^A modification involving METTL3 can affect server classes of RNA splicing, post‐transcriptional modification, and transcription, thereby regulating gene expression level and cellular function. It has been printed out that high‐expressed METTL3 could facilitate the progression of NSCLC through promoting the translation of mRNAs via increasing m^6^A level.^37^ In this study, the increased modification of m^6^A was found in NSCLC cells, and high‐expressed METTL3 could enhance the stability of PHKA1‐AS1. Moreover, METTL3 was found highly expressed in carcinoma tissues compared with adjacent (Figure 6). These results indicated that the increased degree of m^6^A modification enhanced the stability of PHKA1‐AS1 in NSCLC cells and led to increased expression of PHAK1‐AS1, thus promoting the progression and metastasis of NSCLC.

At the same time, there are some limitations in this study. lncRNA PHKA1‐AS1 could bind to ACTN4 and regulate its expression, but the binding sites of PHKA1‐AS1 and ACTN4 have not been revealed. In addition, whether PHKA1‐AS1 regulates ubiquitination by competing with the binding sites of E3 ligase SYVN1 thus disrupting the binding between ACTN4 and SYVN1 remains to be explored. In the future, we will pay attention to the exploration of this molecular mechanism and study the potential value of these molecules in clinical application.

**FIGURE 8:**
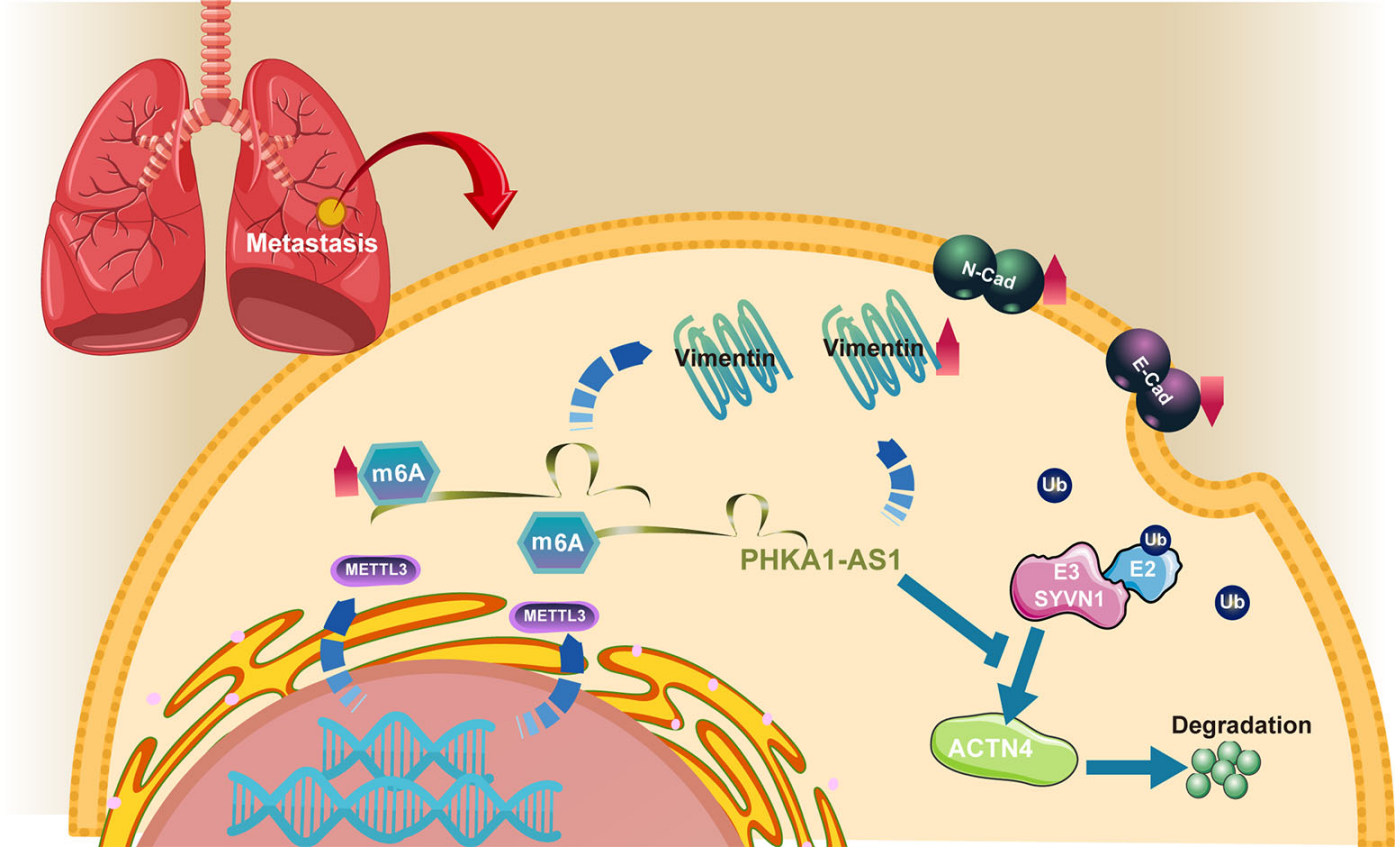
N6‐methyladenosine (m^6^A)‐mediated long noncoding RNA (lncRNA) phosphorylase kinase regulatory subunit alpha 1 antisense RNA 1 (PHKA1‐AS1) promoted proliferation and metastasis of non‐small cell lung cancer (NSCLC) via increasing actinin alpha 4 (ACTN4) stability. The increased m^6^A modification of lncRNA PHKA1‐AS1 could enhance the expression of PHKA1‐AS1 in NSCLC. High‐expressed PHKA1‐AS1 regulated the expression of epithelial‒mesenchymal transition (EMT)‐related proteins and led to the metastasis of NSCLC. In addition, high‐expressed PHKA1‐AS1 could bind to the oncogene ACTN4, inhibited the ubiquitination degradation of ACTN4 and improved its protein stability, thus promoting the progression and metastasis of NSCLC.

In summary, this study has demonstrated that lncRNA PHKA1‐AS1 could facilitate the proliferation and metastasis of NSCLC via increasing ACTN4 stability through regulation of m^6^A modification (Figure 8). These findings provided a theoretical foundation for further investigation into the potential mechanisms of NSCLC metastasis and the identification of new biomarkers associated with NSCLC.

## 4 MATERIALS AND METHODS

### 4.1 Cell culture and reagents

Beas‐2b, A549, H1299, H1975, PC9, H358, and H460 cell lines (iCell Bioscience Inc.) used in this study were all short tandem repeat (STR) identified. Beas‐2b cells were cultured in dulbecco's modified eagle medium (DMEM) medium (Gibco, Invitrogen), A549 cells were cultured in F‐12K medium (Gibco, Invitrogen), and the remaining cell lines were cultured in roswell park memorial institute (RPMI) 1640 medium (Gibco, Invitrogen). All the medium were supplemented with 10% fetal bovine serum (FBS) (Gibco, Invitrogen) and 1% penicillin‒streptomycin (Tianhang Biotech Company).^38^

### 4.2 Preparation of NSCLC tissue samples

Between 2017 and 2019, a total of 32 patients, aged between 38 and 72 years, were admitted to Shanxi Provincial People's Hospital. LUAD tissues were collected from these patients. Among them, 17 were female and 15 were male. None of the patients had undergone chemotherapy prior to surgery. After being treated in phosphate‐buffered saline (PBS) containing 4% paraformaldehyde, the cancer tissue samples were embedded in paraffin for use in immunohistochemical assays. Written informed permission was acquired by each subject. The Ethics Committee authorized and this study was performed according to ethical principles. Both the amount of positively stained cells and the staining intensity were taken into account when assigning a score. The dye color (*a*) intensity was scored as follows: 0 for no color, 1 for pale yellow, 2 for light brown, or 3 for brown. There were four categories for the number of positive cells (*b*): 0 (completely negative), 1 (1%−25%), 2 (26%−50%), 3 (51%−75%), and 4 (>75%). After multiplying the results for (*a*) and (*b*), the specimens were categorized into one of four groups: 0 score (−), 1−4 score (+), 5−8 score (++), and >8 score (+++). Then, the levels were statistically analyzed. In addition, tissue samples from 23 patients with NSCLC were provided by the First Affiliated Hospital of University of Science and Technology of China for tissue RNA extraction.

### 4.3 Transient transfection

Transient transfection includes RNA transfection and DNA transfection. Take the six‐well plate, for example, 20 pmol siRNA and 6 µL RNA TransMate reagent (Sangon Biotech) were added to each well in the RNA transfection, and 5 µg DNA and 10 µL Exfect reagent (Vazyme) were added to each well in the DNA transfection. All the transfection complexes were prepared by serum‐free medium.^39^

### 4.4 CCK‐8 assay

CCK‐8 kits (Dojindo) were utilized to detect the cell viability. Cells (3000‒6000 per well) were spread in 96‐well plates, and the transfection operation was performed after cells attachment. Next, 100 µL of medium and 10 µL of CCK‐8 reagent were mixed and added to each well on the 1st, 2nd, 3rd, and 4th days after transfection. The cell viability was detected by the Bio‐Tek Epoch microplate reader, and the wavelength at 450 nm was used to calculate the absorbance.^40^

### 4.5 Wound healing assay

Horizontal lines were evenly drawn with a mark pen at the bottom of the six‐well plate. Cells were seeded in the well plate and transfected when the cell density reached 80%−90%, and then a pipette tip was utilized to scratch vertically. A microscope was utilized to capture images of different locating points in the plate at 0 and 24 h, respectively. ImageJ was used to count the average width of scratches at each point and the mobility of cells in different groups.^41^ The formula could be expressed as follow: relative motility rate (%) = wound healing area/total surface area × 100.

### 4.6 Transwell assay

Transwell assay includes migration and invasion assay. While pre‐coating was not necessary for the migration assay, Matrigel (Corning) was diluted by serum‐free medium at a ratio of 1:8 to pre‐coat at the upper transwell apparatus chamber. Serum‐free medium containing 5 × 10^4^ cells per well was added to the upper chamber, while 600 µL of 10% FBS‐containing medium was added to the lower chamber. Following a 24‐h culture period, the lower chamber's growth medium was removed, and 500 µL of methanol was added to fix the cells for 30 min before they were stained with 0.5% (w/v) crystal violet. The residual Matrigel and excess crystal violet were wiped gently with a cotton swab in ddH_2_O. Transwell chambers were photographed under a microscope and the number of cells penetrating the membrane was recorded.^42^

### 4.7 EdU assay

In 24‐well plates, cells were planted, and when the cell density reached roughly 70%, they were transfected. The EdU (Sangon Biotech) assay was used to measure the cell proliferation activity after transfection for a duration of 24 h. In accordance with the manufacturer's instructions, the well plate was filled with EdU solution for the ensuing procedures, and pictures of the growing cells were captured utilizing a fluorescence microscope. Both the positive cell rate and the fluorescence intensity were measured.^43^

### 4.8 Colony formation assay

Cells (800 per well) after transfection were inoculated into 12‐well plates until the individual cells grew into colonies with more than 50 cells. Methanol was added for fixation for 1 h, and crystal violet was added for staining for 2 h. After staining, the crystal violet was removed and the remaining crystal violet was gently washed away by ddH_2_O. The plates were put into the scanner for photographing, and the number and size of colony groups were counted.^43^

### 4.9 RNA isolation and real‐time quantitative PCR assay

The RNA extraction kit (Solarbio) was used for extracting total RNA in cells. The PrimeScript RT Master Mix (Takara) was utilized for synthesizing cDNA. The TB Green Premix Ex Taq II (Takara) was executed for real‐time quantitative PCR (RT‐qPCR), and Ct values was detected by LightCycler480 System. GAPDH served as endogenous reference of lncRNA and mRNA. ΔCt = Ct (target gene) ‒ Ct (GAPDH), ΔΔCt = ΔCt (treatment group) ‒ ΔCt (control group), and the relative expression of genes was calculated by formula 2^−ΔΔCt^.^44^ Table S1 displays the primers used in the study.

### 4.10 Western blotting assay

Radio immunoprecipitation assay (RIPA), phenylmethylsulfonyl fluorid (PMSF), and phosphatase inhibitors (Beyotime Biotech) were mixed and added to six‐well plates for cell lysis to extract total protein. The bicinchoninic acid (BCA) protein assay kit (Thermo) was utilized to quantitatively determine the total protein content for every sample. Sodium dodecyl sulfate‐polyacrylamide gel electrophoresis (SDS‒PAGE) was used to isolate protein samples, which were then transferred to a polyvinylidene fluoride (PVDF) membrane. After submerging the PVDF membrane in the blocking solution and shaking it at a medium speed for 1 h at room temperature, the blots were incubated in specific primary antibodies for 12‒16 h at 4°C. After that, the blots were incubated at room temperature with secondary antibodies conjugated with horseradish peroxidase (HRP). Finally, the enhanced chemiluminescence (ECL) solution recognized protein signals (Vazyme).^45^ Table S2 displays the antibodies used in the study.

### 4.11 FISH assay

The corresponding reagents were prepared in accordance with the instructions of the FISH kit (GenePharma). Cells were inoculated in the confocal dish, and the culture medium was discarded after cell adhesion. Then, 4% paraformaldehyde, buffer (A, C, E, F), and 4', 6‐diamidino‐2‐phenylindole (DAPI) solution were added to cells for experimental treatment basing on the manufacturer's instructions. After being washed twice with PBS, the images were observed and photographed under a confocal microscope.^41^ PHKA1‐AS1 probe sequence used was TCCAAGACTTAGGCAATCTGAGAACTGTTGAGACGCAAAGGTATTTAAGCCAACTTTTAAGGCAC.

### 4.12 RNA pull‐down assay

In accordance with the instructions of the Pierce Magnetic RNA‐Protein Pull‐Down kit (ThermoFisher), target RNA with biotin was labeled. The probe‐labeled target RNA was bound to streptavidin magnetic beads, and then incubated with the cell lysate. The RNA‒protein complex was eluted when the complex was formed. Finally, Western blotting assay or mass spectrometry assay was used to detect whether the protein could interact with the target RNA.^40^ Table S2 displays the antibodies used in the study.

### 4.13 Protein silver stain assay

The target protein band obtained in the RNA pull‐down assay was separated by Western blotting electrophoresis. In accordance with the instruction of PAGE Gel Silver Staining Kit (Solarbio), the PAGE gel was silver stained, and the printed PAGE gel was saved as photos.^46^

### 4.14 Lentivirus transfection assay

A549 cells were inoculated into a T75 culture flask, and transfection began when the confluence of cells was about 30%. Lentivirus solution was defrosted on ice, and then 50 µL lentivirus solution (Generalbiol) was added into 10 mL culture medium, and 10 µL polybrene was added to increase transfection efficiency. After 24 h infection, the culture medium was replaced, and the culture was continued for 48 h to stabilize the cell state. Next, the medium with puromycin (MedChemexpress) was added to culture flask until cells on the blank group were killed.^47^

### 4.15 RNA immunoprecipitation assay and m^6^A RNA immunoprecipitation assay

The Magna RIP RNA‐Binding Protein Immunoprecipitation Kit (Millipore) manufacturer's instructions were followed while performing the RIP experiment. An appropriate amount of lysate was prepared and added to the cell precipitation that had been centrifuged. Then, 5 µg immunoglobulin G (IgG) and target antibody were added to the magnetic beads and incubated for 30 min, 10 µL lysate supernatant was collected as the input group, and the remaining lysate, antibody, and beads were mixed and incubated overnight at 4°C rotation. For MeRIP, RNA samples were enriched by the Arraystar Seq‐Star poly (A) mRNA Isolation Kit (Aksomics). After that, anti‐m^6^A antibody‐coated magnetic beads were used to attract RNA samples (Synaptic Systems) or anti‐mouse IgG antibody (Invitrogen). The RNA was subsequently eluted, purified, and finally analyzed by qPCR techniques to explore the interest RNA that bound to the protein.^17^, ^41^

### 4.16 Protein co‐immunoprecipitation assay

Co‐IP experiment was conducted in accordance with the manufacturer's instructions of Pierce Co‐Immunoprecipitation Kit (Thermo). The antibody was immobilized by adding 50 µL coupling resin, 10 µg antibody, 3 µL sodium cyanoborohydride solution, and 200 µL quenching buffer into the centrifuge column in the corresponding steps. Cells were collected by a cell scraper, fully and uniformly mixed with the lysis buffer, and the supernatant after centrifugation was collected. The cell lysates were quantified by a BCA protein assay kit. Then, 50 µL cell lysates of the target protein group and IgG group were taken out as input group and placed at −20°C. The remaining lysates of the target protein group and IgG group were added to the resin containing about 1000 µg crosslinked antibody in each group, which was vortically incubated at 4°C overnight. The protein was then eluted and analyzed by SDS‐PAGE.^48^

### 4.17 Protein stability assay

The over‐expression and knock‐down PHKA1‐AS1 cells were treated with protein synthesis inhibitor cycloheximide (CHX, 100 µg/mL) and incubated for 1, 2, and 3 h, respectively. H1299 cells with knock‐down PHKA1‐AS1 were treated with the proteasome inhibitor MG132 (10 µM). The expression level of ACTN4 was determined using the Western blotting assay.^49^

### 4.18 RNA stability assay

After seeding the cells onto a six‐well plate, the METTL3 plasmid was transfected into the cells until the confluency of the cells reached around 70%. After 24 h of culture, the medium was replaced, and 5 µg/mL actinomycin D (MDBio) was added and incubated for 2, 4, 6, and 8 h, respectively. At last, the total RNA of cells was extracted and the degree of degradation of the target RNA was detected by qPCR.^17^

### 4.19 In vivo assay

All in vivo assays received approval from the Laboratory Animal Center of Guangzhou Medical University and conducted according to the Regulations of Guangdong Province on the Administration of Laboratory Animals and the Regulations of the Experimental Animal Ethics Committee of Guangzhou Medical University. All animals (male BALB/c nude mice aged 4−6 weeks) were purchased from Beijing HFK bioscience Co., Ltd. In each nude mouse, 3 × 10^6^ A549 cells (with a cell suspension of around 200 µL) were injected into the tail vein. Animals were observed every 2 days and body weights were recorded. Four weeks later, 5 nmol si‐NC and si‐PHKA1‐AS1 (Ribobio) were intravenously administered every 2 days to nude mice and body weights were recorded. After continuous injection for 12 times, the animals were sacrificed by dislocating cervical vertebra. The lung tissues were dissected and separated, and then washed by PBS and fixed in 4% paraformaldehyde. After fixation for 24 h, the lung tissues were taken out and photographed for further section and staining.^17^ Routine sections were used for hematoxylin/eosin staining (Servicebio) and IHC analysis. The antibodies used in the IHC include ACTN4 (#DF8000, Affinity) and Vimentin (#5741, CST).

### 4.20 Statistical analysis

GraphPad Prism 8 and SPSS 25.0 were used for all statistical analysis. All experiment results were reported as mean ± standard deviation of three or more repetitions, unless otherwise noted. Using the *t*‐test, one‐way analysis of variance, or rank sum test, the difference between the groups was ascertained. The association was evaluated using Pearson's correlation method. *p*‐Values below 0.05 were considered statistically significant.

## AUTHOR CONTRIBUTIONS

Q.G. conceived and designed the experiments and performed the experiments. G.Z. wrote the paper and contributed data. W.Z. performed the experiments and analyzed and interpreted the data. Y.L. contributed reagents and analyzed and interpreted the data. X.C. and Z.D. performed the experiments. J.L. contributed analysis tools. H.B. and M.W. contributed materials. M.X. contributed reagents. Y.Y. analyzed and interpreted the data and wrote the paper. J.Z. conceived and designed the experiments and contributed reagents, materials, analysis tools, or data. All authors have read and approved the final manuscript.

## CONFLICT OF INTEREST STATEMENT

The authors declare they have no conflicts of interest.

## ETHICS STATEMENT

Animal ethics approval is approved by the Laboratory Animal Center of Guangzhou Medical University (number: GY2020‐112). The Ethics Committee of Shanxi Provincial People's Hospital approved the study (number: 20230042). Medical Research Ethics Committee of the First Affiliated Hospital of University of Science and Technology of China approved the study (number: 2023‐RE‐344).

## Supporting information

Supporting Information

## Data Availability

All data used and analyzed in this study are available from the corresponding author upon reasonable request.
