# Supplementary material for: m6A modification of lncRNA PHKA1‐AS1 enhances Actinin Alpha 4 stability and promotes non‐small cell lung cancer metastasis
Source: MedComm (2020). 2024 May 17;5(6):e547. doi: 10.1002/mco2.547 (PMC11099756; doi:10.1002/mco2.547)
Supplement: Supplementary file 1 — Supporting Information [file MCO2-5-e547-s001.docx]

**m^6^A modification of LncRNA PHKA1-AS1 enhances ACTN4 stability and promotes non-small cell lung cancer metastasis**

Qiao-ru Guo^1#^, Guo-bin Zhang^1#^, Wen-min Zhou^1#^, Yu Lu^1#^, Xin-zhu Chen^1^, Zhuo-fen Deng^1^, Jin-shuo Li^2^, Hong Bi^3^, Ming-sheng Wu^4^, Ming-ran Xie^4^, Yan-yan Yan^2*^, Jian-ye Zhang^1, 5*^

^1^Guangzhou Municipal and Guangdong Provincial Key Laboratory of Molecular Target & Clinical Pharmacology, the NMPA and State Key Laboratory of Respiratory Disease, School of Pharmaceutical Sciences and the Fifth Affiliated Hospital, Guangzhou Medical University, Guangzhou 511436, P.R. China

^2^School of Medicine, Shanxi Datong University, Datong 037009, P.R. China

^3^Department of Pathology, Shanxi Provincial People’s Hospital, Taiyuan 030012, P.R. China

^4^Department of Thoracic Surgery, The First Affiliated Hospital of USTC, Division of Life Sciences and Medicine, University of Science and Technology of China, Hefei, 230031, P.R. China

^5^The Affiliated Qingyuan Hospital, Guangzhou Medical University, Qingyuan 511518, P.R. China

^#^These authors contributed equally to this work and shared first authorship.

^*^Correspondence: Jian-ye Zhang (Email: [jianyez@163.com](mailto:jianyez@163.com));

Yan-yan Yan (Email: [zwsanyan@163.com](mailto:zwsanyan@163.com)).

Table S1 The primers/siRNA/Probe used in the study

| Primer/siRNA/Probe | Sequence |
| --- | --- |
| PHKA1-AS1 F | CTGAATTGTACTGGTCTGTCCT |
| PHKA1-AS1 R | GCTTAGATGTGGTGCTGTGG |
| GAPDH F | GAAGGTGAAGGTCGGAGTCAACG |
| GAPDH R | TGCCATGGGTGGAATCATATTGG |
| Si-PHKA1-AS1  *In vivo* si-PHKA1-AS1 | GCGTCTCAACAGTTCTATA  GCGTCTCAACAGTTCTATA |
| PHKA1-AS1 FISH Probe | TCCAAGACTTAGGCAATCTGAGAACTGTTGAGACGCAAAGGTAGTTTAAGCCAACTTTTAAGGCAC |
| PHKA1-AS1-26A F | ATCCTGTGCCTCCTGTAGAGC |
| PHKA1-AS1-26A R | ATCCTGTGCCTCCTGTAGAGC |
| PHKA1-AS1-56A F | TGCTTGTCGAACAAAGAATGC |
| PHKA1-AS1-56A R | CATCTGTGCCATTCAACATCTTC |
| PHKA1-AS1-214A F | AAATGAAGATGTTGAATGGCA |
| PHKA1-AS1-214A R | CAAGACTTAGGCAATCTGAGC |
| PHKA1-AS1-246A F | GAGCTCAGATTGCCTAAGTCTTG |
| PHKA1-AS1-246A R | ATACCAGCCATTTTCTCCAGTG |
| METTL3 F | GAACAACAGAGCAAGAAGGTCAGTC |
| METTL3 R | CTCCTCCTTGGTTCCATAGTCACAG |
| Si-ACTN4  Si-MARCH1 | GCAGCAUCGUGGACUACAA  GAGAAGAACUUCUCAUGUA |
| Si-MARCH6 | GCGGAUUUAUCCAUUUUUA |
| Si-SYVN1 | CCUACUACCUCAAACACCA |

Table S2 Antibody

| Antibody | Dilution ratio | Company |
| --- | --- | --- |
| GAPDH (MB001) | 1:10000 | Bioworld |
| β-actin (AF7018) | 1:2000 | Affinity |
| E-Cadherin (3195) | 1:1000 | Cell Signaling Technology |
| N-Cadherin (13116) | 1:1000 | Cell Signaling Technology |
| Vimentin (5741) | 1:1000 | Cell Signaling Technology |
| ACTN4 (ab108198) | 1:1000 | Abcam |
| Goat Anti-Rabbit IgG (H+L) HRP (S0001) | 1:5000 | Affinity |
| Goat Anti-Mouse IgG (H+L) HRP (S0002) | 1:5000 | Affinity |
| METTL3 (ab195352) | 1:1000 | Abcam |
| SYVN1 (13473-1-AP) | 1:1000 | Proteintech |
| Anti-m6A (202111) | 5 μg/mL | Synaptic Systems |
| IgG (11203D) | 1μg/μL | Invitrogen |

PHKA1-AS1

NCBI Reference Sequence: NR_110391.1 (<https://www.ncbi.nlm.nih.gov/nuccore/NR_110391.1>)

Sequence: ATCCTGTGCCTCCTGTAGAGCCCAGACACTGCTTGTCGAACAAAGAATGCAGAGGACCGTAGAGAGCAGTCTACAGCCTGAGACCCATCATTATGGCCTGGCACATGATGATTACACAGTCTTTTAAATTTAAAAAAATGAAGATGTTGAATGGCACAGATGAATTCTCTTCAATTCTCTGACATTATTTCTCAGAAAGAAACCAAGATGAAGACTGGAGCTCAGATTGCCTAAGTCTTGGAGAGACCACTGGAGAAAATGGCTGGTATAGAGAGAATTTTTCCCACTAATGGAAATGAATGCACATGCATCTTATAACTGACAACAGAGTACTTCAACTCCCACCATAGCCCAGAATATTGTTTTCTTAATATTATTTTCTTGGTCGTTGAAGAGGATGCAGCTACCTTTGCGTCTCAACAGTTCTATAAAGGATGCTGGTTTACTGAGGGGAAGTGCCTTAAAAGTTGGCTTAAAGGCATCTGCTGACTCTGACCTCATTTTGCCCAGGAGTTGGGGGAGGGAGGAAGCAACAGGCTCTGAAACAGTGGCCACAGCTATAAAAATCAACAGATAAAGTCTTTACTGTGTCTAGTCCA


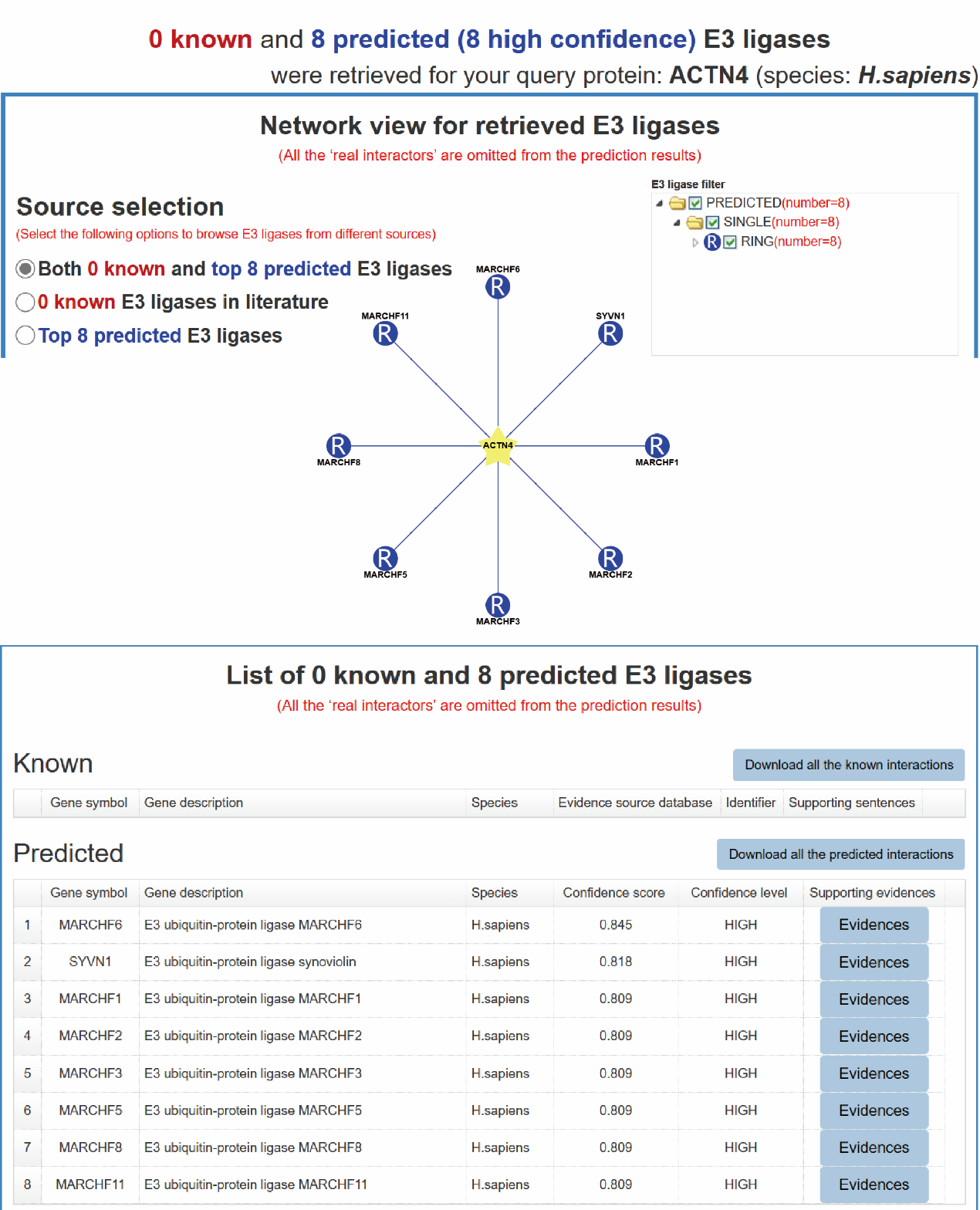


**Figure S1. UbiBrowser 2.0 database (http://ubibrowser.ncpsb.org.cn) predicted the E3 ligases that are likely to bind to ACTN4.**


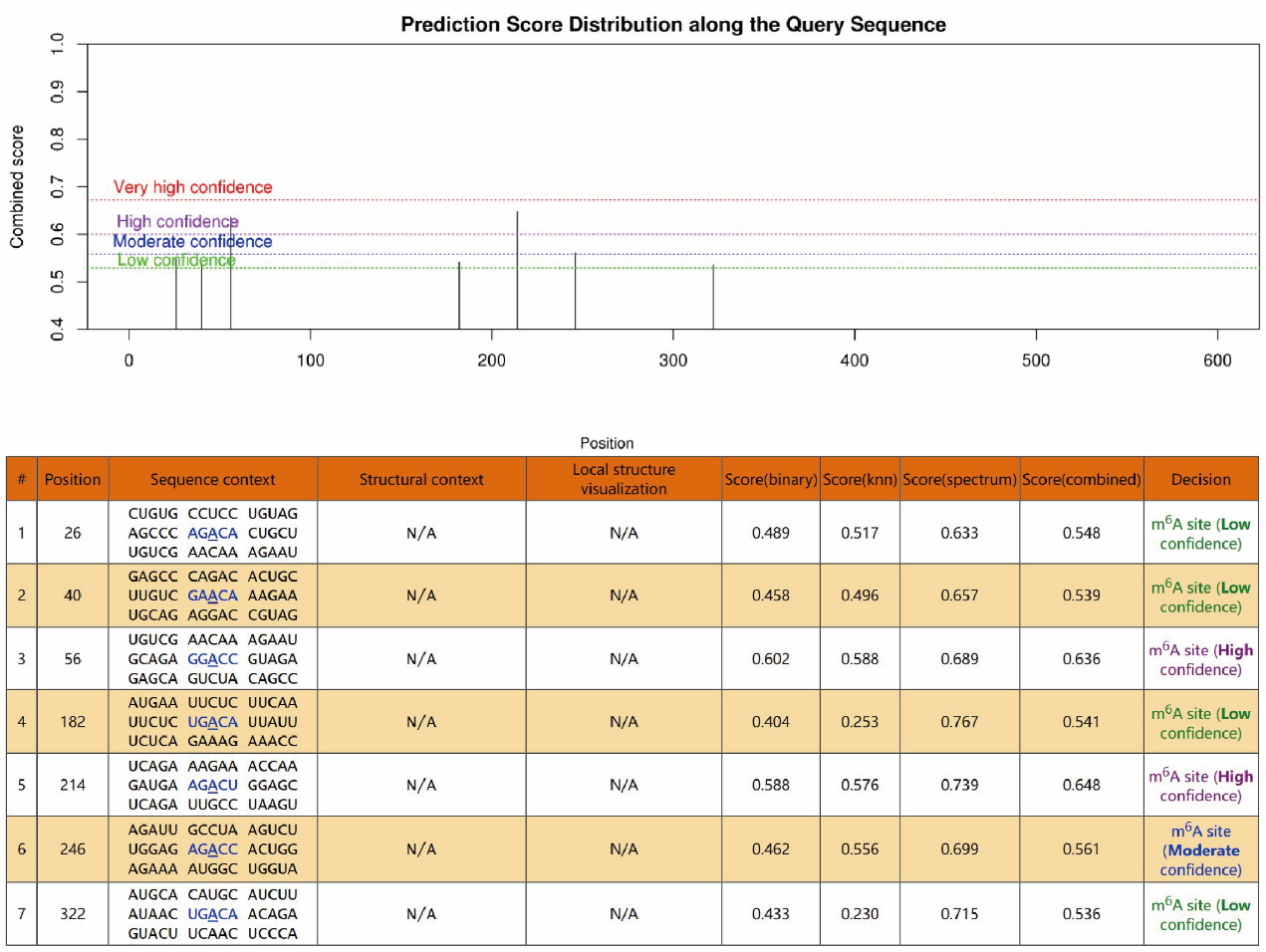


**Figure S2. SRAMP algorithm (**[**http://www.cuilab.cn/sramp**](http://www.cuilab.cn/sramp)**) was utilized to predict the m^6^A binding sites of PHKA1-AS1.**
